# The Medical Virtues of the Compound Fluid Extract of Tephrosia Virginiana

**Published:** 1855-10

**Authors:** B. O. Jones

**Affiliations:** Atlanta, Georgia


ARTICLE V.—*The Medical Virtues of the Compound Fluid Extract of Tephrosia Virginiana.* BY B. O. JONES, M. D., Atlanta, Georgia.

Fully impressed with the many improvements and discoveries of the age, both as regards science and literature, as well as every other department of life, it would scarcely be expected that one so humble as myself should bring much that is important to the shrine of medical literature at the present day; yet, when we reflect that age after age—from the days of Æsculapius down to the present time—have each brought in their contributions to swell the volumes of medical literature, and that discovery after discovery, the result of thorough and scientific investigation, have greatly astonished the world, still may we be permitted to hope that the end is not yet, and that the half has not yet been told, and that the "great healing art" shall take her position co-equal with the wonderful advancement of other departments of knowledge, and with healing on her wings give health and comfort to the afflicted of every land and every clime beneath the sun.

Under such impressions as these, I have ventured to introduce to the medical world the result, to some extent, of my experience in the treatment and cure of disease with the Compound Fluid Extract of Tephrosia Virginiana, or, as I have more commonly called it, in honor of the first discoverers, "Indian Sarsaparilla;" and if, on farther investigation, it shall meet with the approbation I contemplate it will, I shall feel I have discharged an obligation, long resting upon me, in giving the history and virtues of this plant to the profession.

Tephrosia Virginiana is of the order Leguminosa, Sub Ord Papilionacea, a well known indigenous perennial plant, growing abundantly throughout most of the Southern States, putting up early in the spring, growing to the height of eighteen or twenty inches, flowers in June and July, and attains its maturity in August and September. The whole plant is medicinal, and should be taken up by the root in early summer, whilst in full bloom and carefully dried in the shade.

Preparation of Compound Fluid Extract Tephrosia Virginiana:

Tephrosia Virginiana,........................................8 ounces.
Rumex Acutus..................................................2 "
Aqua Font,......................................................4 quarts.

Boil down to one quart.

With the above mixture prepare the following for use, which will keep for any length of time without injury:

Comp. Fluid Ext. Teph. Virginiana.................4 ounces
Alcohol Dilutum, (Brandy,)...........................4 "
Saccharum Album..........................................2 "

Mix and digest for several days, and then strain through muslin, and it is ready for use. Dose for an adult from one-half to one fluid ounce, repeated as often as symptoms demand, with a less dose in proportion for children. It should be given at all times with a view to its tonic and stimulant effect, and continued for some time. I have seen results from this medicine, or from other unknown causes, nature perhaps, truly astonishing; so much so, that I shall forbear to mention them, preferring that others should test its virtues also, and decide accordingly, receiving only the genuine coin and admitting only that which has been unmasked, weighed in the balance, and amply sustained by tangible data.

I will venture, however, to mention in this connection a fact well known to every practitioner of medicine, that there is a time in the treatment of many diseases, and especially Typhoid fever, when there can be little if any use for active medicine—the patient not yet sufficiently recovered to dispense with medicine altogether—when one imprudent act of the physician will prove almost fatal; still it is of the greatest importance that something should be done to perfect the cure already commenced. Some mild, stimulating tonic, having a slight action on the bowels and the secretive organs generally, would seem to meet the indication. This, we feel authorized to say, will be accomplished as well by the use of this medicine as any other that ever came to our knowledge.

It may not be improper, indeed I think it necessary, to mention a few important cases treated mainly with this medicine:

Case 1.—Fanny, a colored woman, the property of N. M., of Coweta County, Georgia, had been sick with general dropsy for some eight or ten months, occasioned, in all probability, by Intermittent fever, attended with enlargement of the liver and spleen, together with obstruction of the catamenial discharge. Had been treated with the usual remedies in such cases, to wit: Cathartics, Diuretics, and Tonics, to which may be added mercurials, iodine, &c.; indeed all the common medicines recommended by the books of the day for the cure of those diseases, with but little if any effect. The disease continued gradually to get worse, until it assumed strong indications of a fatal result, and thus remained for several days, when I became satisfied if something more was not done, and that speedily, the patient would surely die. I then mentioned the fact to the owner of the woman and requested consultation, to which he seemed unwilling, saying at the time he had no hopes of her recovery now or for some time past, and that he thought it best to let the woman die in peace; besides he was unable to defray the expenses of such policy, and that he preferred my continuing in the case to the end, and if I could see at any time that there was anything possible to be done, to fill the indication at once. I mentioned that the Indians used the Tephrosia Virginiana in the treatment of dropsy with seeming good effect, and proposed its use in this case, to which he readily assented. The patient was immediately put under the full influence of this medicine, and kept so for several days, when the symptoms became greatly improved, and in the course of five or six weeks was almost entirely cured.

CASE 2.—A. M., aged 25 to 30 years, of Fayette County, Georgia, Typhoid fever.

*Symptoms.*—Pain and stiffness in the back of the neck, extending down the left hypochondriac and lumbar region. Pulse 120 to 130. Skin of head and upper extremities hot and dry; inferior extremities cold and shriveled; a dark brown incrustation upon the tongue, and the teeth aud gums covered with sordes. Had been confined some eight or ten days; had taken no active medicine, or could be prevailed upon to do so, however was induced by my suggestion to use the Compound Fluid Extract of Tephrosia Virginiana, which he continued with a few other mild remedies, such as friction and sinapisms to the spine and extremities, until a cure was effected.

CASE 3.—Philip, a black boy, aged some 18 or 20 years, had rheumatism of several years' standing. Left inferior extremity useless, stiff and emaciated. General health impaired; subject to occasional acute attacks in other parts of the system, attended with much pain, swelling and fever. Put him under treatment with use of the Compound Fluid Extract of Tephrosia Virginiana, and continued with little if any other medicine for some two or three months, when I had the satisfaction of finding the disease entirely removed, and he has been well, with the exception of a slight stiffness in the hip joint, ever since.

Thus will be seen, I hope, sufficient to cause at least a farther investigation, and to the ordeal of critical analysis and experiment these observations are submitted, with a desire upon my part that the truth should be evolved, whether the virtues of the article, which I have brought to the notice of the profession, shall rise or fall.

